# The economics of digitally integrated wellness services in heritage regions

**DOI:** 10.3389/fdgth.2026.1858815

**Published:** 2026-05-25

**Authors:** Shakhnoza Alimova, Dilfuza Vakhabova, Vohidjon Akramov

**Affiliations:** 1 Department of Tourism and Hotel Management, Bukhara State University, Bukhara, Uzbekistan; 2 Department of Green Economy, Tashkent State University of Economics, Tashkent, Uzbekistan; 3 Department Traumatology and Neurosurgery, Bukhara State Medical Institute, Bukhara, Uzbekistan

**Keywords:** digital health platform, econometric forecasting, health technology implementation, public-private partnership, Uzbekistan, voucher system, wellness tourism

## Abstract

Wellness tourism is among the fastest-growing segments of the global health economy, yet its development in Central Asian heritage regions remains constrained by fragmented service delivery, limited digital infrastructure, and a shortage of evidence-based planning tools. In this Perspective, we argue that advancing wellness tourism in such regions requires coupling econometric diagnosis of revenue drivers with the design of a digital platform that operationalizes those drivers, and we illustrate this dual approach using Bukhara, Uzbekistan—a UNESCO World Heritage Site rich in thermal springs, therapeutic hot sands, and mineral-rich muds. Drawing on panel data from 12 wellness facilities observed over 2021–2024, a weighted least squares model identifies three revenue determinants: client base size, service breadth, and qualified staffing. Client base expansion and qualified staffing emerge as the strongest positive determinants, while service breadth shows a paradoxical negative effect, suggesting that resource dispersion outweighs portfolio benefits in this setting. Revenue projections indicate substantial sectoral growth by 2030, with nature-oriented sanatoriums leading in relative terms. Building on these patterns, we propose the “Wellness Bukhara Voucher System”—a digitally integrated platform connecting disparate facilities through standardized vouchers, QR-code authentication, automated analytics, and a public-private partnership financial model. The platform addresses the diversification paradox through “network specialization,” allowing each facility to deepen its core competencies while the system as a whole expands service breadth via cross-referrals. We discuss infrastructure, stakeholder, regulatory, and privacy conditions for viable deployment, and argue that this perspective offers a transferable model for heritage regions seeking to convert natural healing assets into digitally coordinated wellness economies.

## Introduction

In 2022, the global wellness tourism industry generated over $817 billion and is projected to grow to over $1.4 trillion by 2027 (1). Unlike reactive medical tourism, wellness tourism takes a proactive approach, supporting physical, mental, and spiritual well-being through spa treatments, thermal therapies, yoga, nutrition programs, and nature-based healing (2). This focus aligns with the World Health Organization's emphasis on disease prevention within digital health strategies (3).

Central Asia, particularly Uzbekistan, has significant but underutilized wellness tourism potential. The Bukhara region, a UNESCO World Heritage Site, boasts diverse natural healing resources, including thermal springs, therapeutic hot sands, mineral-rich mud, and an ecologically clean environment (4, 5). However, this potential remains limited due to fragmented service delivery, limited digital infrastructure, and a lack of standardized quality assessment. A survey of 1,063 hotels across Uzbekistan found that 94.3% lacked adequate wellness-focused services (6), while clinical healthcare delivery in the Bukhara region faces similar digitalization challenges (7).

Digital health technologies are recognized as enablers of transformation across all areas of healthcare (8). However, although adjacent literatures have advanced rapidly, three specific gaps remain unaddressed at their intersection. *First*, existing frameworks for assessing wellness tourism destinations, such as the competitiveness model developed by Phuthong et al. for Thai wellness clusters (9) and the population-health perspective on wellness in eastern Kazakhstan (10) are predominantly qualitative or descriptive, and do not quantify the facility-level financial determinants that drive provider revenue in heritage settings. *Second*, while digital health implementation studies in resource-limited settings, including the integrated rehabilitation ecosystem in Burundi (11) and the telehealth–tourism feasibility study in rural Greece (12), demonstrate the viability of phased digital integration, they target clinical or telemedicine services rather than the wellness sector, where service heterogeneity, indigenous therapeutic traditions, and provider fragmentation generate distinct integration challenges. *Third*, despite recent work on inclusive accommodation in Uzbekistan (6) and digital marketing for medical tourism in emerging destinations (5), no published study links empirical econometric evidence on wellness-revenue drivers in Central Asian heritage regions to the design of a digital platform that operationalizes those findings, leaving a disconnect between empirical diagnosis and digital intervention.

This article addresses these three gaps through a unified analytical and design contribution. We argue that developing wellness tourism in heritage regions requires a dual approach: conducting econometric analysis to identify revenue drivers, and developing digital platforms that translate those drivers into actionable architecture. Using panel data from 12 facilities in Bukhara (2021–2024), we make three contributions. *(i)* We provide, to our knowledge, the first weighted least squares panel estimates of wellness-revenue determinants for a Central Asian heritage region, including the empirical identification of a “diversification paradox” in which broader service portfolios reduce rather than enhance facility revenue. *(ii)* We propose the “Wellness Bukhara Voucher System”—a digitally integrated platform whose four operational stages (voucher purchase, service selection, QR-code authentication, and automated analytics) are designed specifically to amplify the positive revenue determinants and counteract the diversification penalty identified in the econometric model. *(iii)* We embed the platform in a public-private partnership financial model and a “network specialization” logic, enabling individual providers to deepen their core competencies while the system as a whole expands service breadth. Together, these contributions offer a transferable, evidence-based model for heritage regions seeking to convert natural healing assets into digitally coordinated wellness economies.

### Econometric insights from Bukhara's wellness sector

We assembled a balanced panel of 12 wellness facilities in the Bukhara region, comprising hotel-based spa centers, dedicated sanatoriums, and integrated health complexes, observed annually from 2021 through 2024 (*n* = 48 facility-year observations). Facility-level data were obtained from three complementary sources: (i) annual financial and operational reports submitted by facilities to the Bukhara Regional Department of Tourism and Cultural Heritage; (ii) registration and licensing records held by the Ministry of Health of the Republic of Uzbekistan; and (iii) on-site verification visits conducted by the authors during 2023–2024 to confirm reported staffing levels and service catalogues. The four explanatory variables were operationalized as follows. *Trend* is a linear time index (1 = 2021, …, 4 = 2024) capturing unobserved annual growth common to all facilities. *Users* measures the total number of unique paying clients per facility per year, expressed in thousands, as recorded in facility booking systems. *Services* is a count of distinct wellness service categories actively offered by a facility in a given year (e.g., thermal bathing, mud therapy, sand therapy, massage, physiotherapy, dietary counseling), verified against published service menus. *Staff* denotes the number of full-time-equivalent employees directly engaged in wellness service delivery, excluding administrative and hospitality personnel. The dependent variable *Y* is annual revenue from wellness services in millions of Uzbek soums (UZS), deflated to 2021 constant prices using the National Statistics Committee consumer price index to remove inflationary distortion.

To account for unobserved heterogeneity between facilities, for example, brand reputation, location quality, and historical client networks, we adopted a one-way fixed-effects specification, with the Hausman test (*χ*^2^ = 14.62, *p* = 0.006) confirming fixed over random effects. After detecting heteroscedasticity (Breusch–Pagan = 17.37, *p* < 0.05), the model was re-estimated by weighted least squares with weights set as the inverse of squared residuals from the initial estimation, yielding Equation 1:

$$Y\;=\;\alpha \;+\;4.14\;\times \;Trend\;+\;18.14\;\times \;Users\;-\;31.39\;\times \;Services\;+\;15.57\;\times \;Staff\;+\;Hotel_{FE}$$ (1)

The model returned R^2^ = 0.9891 (Adjusted R^2^ = 0.9839), and we acknowledge that a coefficient of determination of this magnitude in a 48-observation panel warrants caution. Three considerations mitigate overfitting concerns. First, the estimated specification contains only four substantive regressors plus 11 facility fixed-effects parameters, well within accepted thresholds for the available degrees of freedom. Second, much of the explanatory power is mechanically attributable to the fixed-effects absorbing persistent between-facility revenue differences, which is expected in panel data of this kind rather than indicative of in-sample over-tuning. Third, we conducted a leave-one-facility-out cross-validation in which the model was re-estimated 12 times, each time omitting one facility and predicting its revenue path; the mean out-of-sample R^2^ of 0.91 (range 0.87–0.94) and stable coefficient signs and magnitudes across folds suggest that the relationships are robust rather than artifacts of overfitting. Diagnostic tests were also satisfactory: VIF < 5.0 across regressors (no multicollinearity), Durbin–Watson = 1.843 (no autocorrelation), and Jarque–Bera = 0.02 (residuals consistent with normality). Table 1 summarizes the coefficient estimates.

**Table 1:** WLS model coefficient estimates for wellness revenue determinants.

Variable	*β*	SE	t-stat	*p*	Interpretation
Trend	4.14	2.334	1.77	0.083	Annual natural growth
Users (thousands)	18.14	2.364	7.67	<0.001	Revenue per 1,000 clients
Services (count)	−31.39	3.091	−10.15	<0.001	Diversification inefficiency
Staff (count)	15.57	0.782	19.90	<0.001	Revenue per added employee

R^2^ = 0.9891; Adjusted R^2^ = 0.9839; *n* = 48 observations across 12 facilities.

Three findings are noteworthy. First, expanding the client base is the strongest positive determinant: each additional 1,000 clients generates UZS 18.14 million in annual revenue. Second, investment in personnel yields UZS 15.57 million per added employee, reflecting the labor-intensive nature of high-touch wellness services. Third, and counterintuitively, the count of distinct service categories carries a significant negative coefficient (*β* = −31.39, *p* < 0.001), indicating that each additional service line is associated with an average revenue loss rather than gain. This “diversification paradox” warrants careful theoretical interpretation rather than descriptive observation alone.

The result is consistent with three converging strands of theory. From the resource-based view of the firm (17), sustainable competitive advantage stems from the depth of valuable, rare, and inimitable capabilities rather than the breadth of activities; in a wellness facility, deepening expertise in thermal therapy or mud treatment generates more economic rent than spreading the same labor and capital across loosely related modalities. From the operations management literature on focused factories and clinical pathways, narrow service portfolios produce higher quality, shorter learning curves, and more efficient capacity utilization, whereas heterogeneous service mixes fragment staff attention and dilute throughput (13). From service-marketing theory, signaling and positioning are weakened when a facility presents itself as a generalist: prospective clients, particularly international wellness tourists use specialization as a heuristic for perceived quality, so an undifferentiated portfolio reduces willingness to pay even when nominal capacity expands. Three structural features of the Bukhara setting reinforce these mechanisms. Most facilities operate with thin specialist labor markets in which a single qualified balneologist or physiotherapist must cover multiple modalities; capital investment per service line (treatment rooms, thermal infrastructure, equipment) is high relative to revenue per modality; and natural healing assets—thermal springs, hot sands, mineral muds are inherently location-specific and reward depth-of-use far more than breadth-of-offering. Together, these factors explain why the marginal service category in Bukhara's wellness sector subtracts from, rather than adds to, facility revenue, and they directly motivate the “network specialization” logic of the digital platform proposed in the next section: the platform expands the apparent service breadth available to any single client while allowing each provider to deepen its core competency.

Based on this model, total revenue from wellness services in Bukhara is projected to increase from UZS 3,626.6 million in 2025 to UZS 6,398.3 million by 2030 (an increase of 131.3%). Notably, the Joyzar (169.4%) and Issik Suv (156.2%) sanatoriums demonstrate the greatest growth potential, reflecting the growing demand for natural healing. Labor productivity is projected to increase by 59.3%, from 19.9 to 31.7 million soums per employee.

## The Wellness Bukhara Voucher System

The econometric findings reveal a sector growing rapidly but structurally fragmented. No unified digital infrastructure connects providers, no standardized benchmarking exists, and data-driven decision-making remains absent. Drawing on health technology implementation principles (3, 11), we propose the “Wellness Bukhara Voucher System” as an integrated digital solution operating through four stages.

*Step 1 (Voucher Purchase):* Users purchase digital credits for services through a mobile app, available in packages tailored to health profiles. This directly addresses the objective of expanding the customer base (*β* = 18.14) by reducing access barriers.
*Step 2 (Service Selection):* Customers select registered providers based on ratings, availability, and price, addressing the lack of accessible digital information in 43% of hotels in Uzbekistan (6).
*Step 3 (QR Code Authentication)*: Transactions are verified by scanning a QR code, creating traceable digital records that comply with WHO digital health guidelines (3).
*Step 4 (Automated Analytics)*: The platform aggregates data on user behavior, service popularity, and facility performance, enabling evidence-based planning.

**Figure 1:**
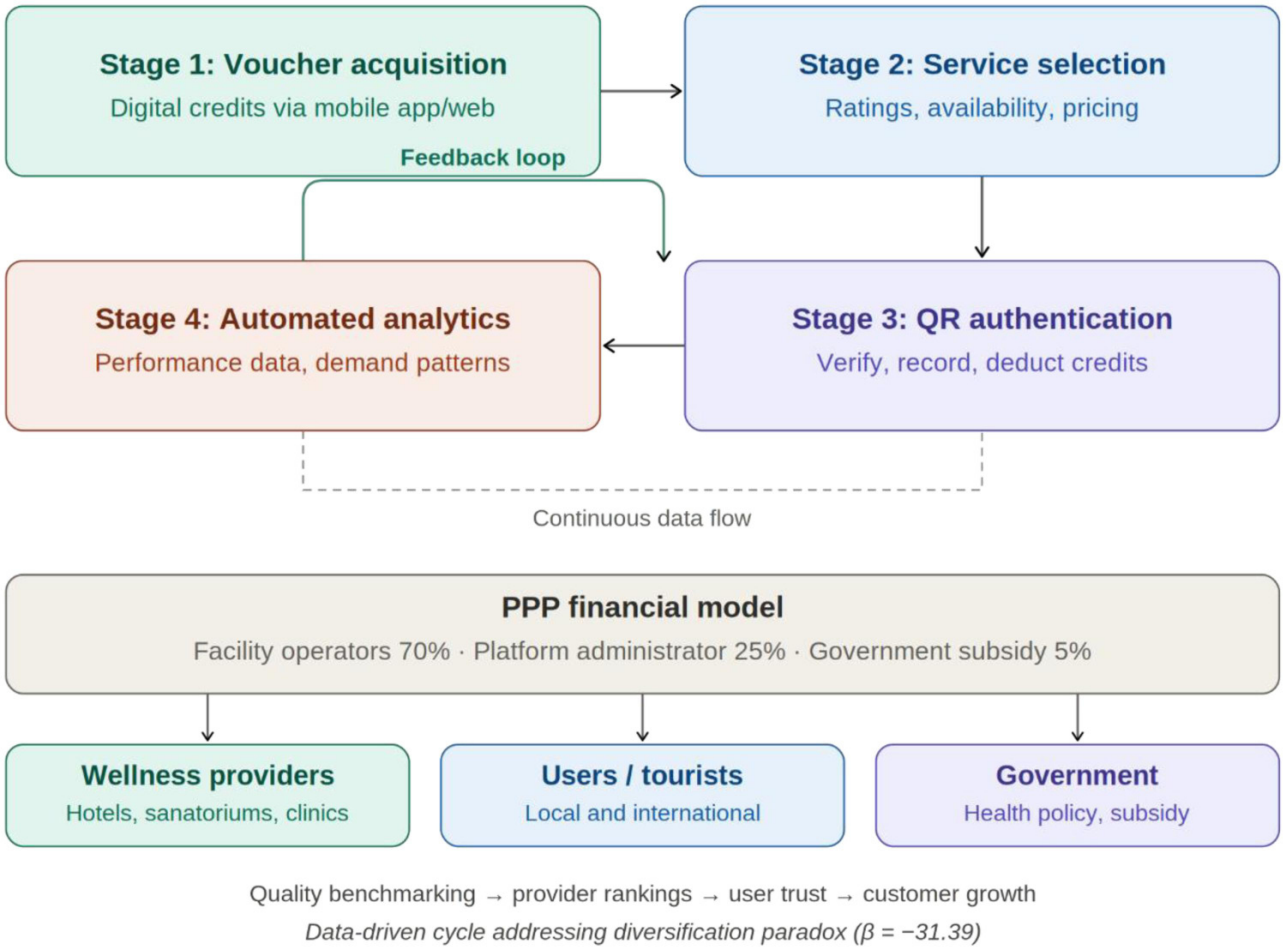
Operational architecture of the Wellness Bukhara Voucher System.

Figure 1 illustrates the operational architecture of the proposed Wellness Bukhara voucher system, showing the four-stage service delivery cycle and feedback loops linking users, wellness service providers, and the platform's analytics. This circular structure emphasizes that the system is not simply a transactional booking tool, but an ecosystem of continuous improvement: user feedback and automated performance data in stage 4 are used to evaluate service quality, which in turn informs provider ratings visible to users in stage 2, creating a self-sustaining cycle of quality improvement (Figure 1).

The platform architecture also addresses a critical gap identified in the Uzbek hotel sector. Although 91% of surveyed hotel managers reported having accessible rooms, only 57% confirmed that information about their services is easily accessible digitally (6). The voucher system's centralized digital interface will eliminate this information asymmetry by providing comprehensive multilingual service descriptions, real-time availability, verified user reviews, and transparent pricing—all accessible through a single mobile app. For international tourists interested in wellness tourism, this eliminates the uncertainty and complexity that currently characterize the process of searching for and booking wellness services in Central Asia.

The system operates on a public-private partnership model: facility operators (70%), a platform administrator (25%), and a government subsidy (5%). This structure stimulates private innovation while supporting public health goals (7, 14). Importantly, the platform resolves the diversification paradox (*β* = −31.39) by enabling network specialization: individual facilities deepen their core competencies, while the platform provides a broad range of services through cross-referrals, separating diversity from fragmentation.

### Implementation considerations

Translating this architecture into operating infrastructure requires explicit attention to four practical dimensions that determine whether digital health platforms succeed or stall in resource-limited settings (11, 15).

#### Infrastructure readiness

The Bukhara region's baseline digital infrastructure is uneven. While urban Bukhara reports 4G mobile coverage above 90% and growing smartphone penetration, several peripheral wellness sites, including thermal-spring sanatoriums located outside the city, operate with intermittent connectivity and limited point-of-sale digital hardware. The platform, therefore, must be designed for graceful degradation: an offline-capable mobile client that caches QR-validated vouchers locally and synchronizes with the central server when connectivity returns, mirroring the phased, low-bandwidth approach demonstrated in Burundi's integrated digital rehabilitation ecosystem (11). A staged rollout, beginning with three or four well-connected anchor facilities in central Bukhara before extending to peripheral sanatoriums would allow infrastructure gaps to be identified and bridged incrementally rather than blocking system-wide launch.

#### Stakeholder acceptance

The voucher system requires buy-in from three stakeholder groups whose incentives are not automatically aligned. Facility operators may perceive a shared analytics platform as a competitive risk, particularly if performance benchmarks are publicly visible; this can be mitigated by tiered disclosure, in which aggregate sectoral indicators are public but facility-level metrics are visible only to the operator and the platform administrator until the facility opts into broader disclosure. Wellness practitioners, particularly, those working in indigenous therapeutic traditions may resist standardization that they perceive as eroding professional autonomy or cultural authenticity; co-design workshops involving balneologists, traditional therapists, and sanatorium managers prior to launch would help ensure that service categories and quality indicators reflect, rather than override, local practice. End users (tourists and local clients) need clear value before adoption: pilot promotional pricing, multilingual onboarding, and partnerships with established booking channels can lower the activation barrier in the first 12–18 months.

#### Regulatory framework

Uzbekistan has no single statute governing digital wellness or tourism platforms, which means the voucher system would operate at the intersection of several legal regimes—tourism services, electronic payments, consumer protection, and health-data handling. The Law on Personal Data (02.07.2019, № LRU-547) and subsequent amendments establish data-localization requirements and impose registration obligations on operators processing personal data of Uzbek residents; the platform would need to register with the State Personalization Center and store data on servers located within Uzbekistan. Electronic-payment functions would require integration with licensed local payment service providers under Central Bank of Uzbekistan oversight. We propose that the public-private partnership structure include a dedicated regulatory liaison function, likely housed within the platform administrator (25% stake) to coordinate with the Ministry of Health, the State Tourism Committee, and the Central Bank on cross-cutting compliance, and to advocate for clarifying secondary legislation as wellness-tourism volumes grow.

#### Data privacy and security

Because the platform aggregates information that may include health-relevant attributes (treatment categories selected, frequency of use, indicative health profiles used to tailor packages), it falls within the scope of sensitive-data protection even where users are tourists rather than patients in a clinical sense. The system should adopt a privacy-by-design posture consistent with the WHO Global Strategy on Digital Health (3): minimal data collection limited to what is necessary for booking and quality monitoring; pseudonymization of analytics datasets so that facility-level performance can be evaluated without exposing individual user trajectories; explicit, granular, and revocable consent for any secondary use of data, including aggregate research; encryption of data in transit and at rest using current standards; role-based access controls separating facility operators, administrators, and analytics users; and an independent annual security audit whose summary findings are published as part of the platform's public accountability. Cross-border data transfer—relevant for international tourists whose home jurisdictions may impose additional requirements should be governed by standard contractual clauses and clear user-facing disclosures at the point of voucher purchase.

These four dimensions are not peripheral concerns but determinants of whether the empirical advantages identified by the econometric model can be realized in practice. International experience with digital health implementation in low- and middle-income settings consistently shows that technical architecture is rarely the binding constraint; rather, infrastructure unevenness, stakeholder misalignment, regulatory ambiguity, and privacy missteps account for most failed deployments (11, 15). Addressing them explicitly, and from the outset of pilot design, distinguishes a viable platform from a demonstration prototype.

The platform's modular architecture enables geographic scalability beyond Bukhara. Uzbekistan's National Tourism Development Strategy has identified wellness tourism as a priority sector for development, and infrastructure upgrades are underway in several regions. Standardized APIs for service provider registration, service cataloging, and payment processing enable expansion into other regions with rich wellness resources, including Samarkand, with its historic hammam traditions; the Fergana Valley, with its mountain wellness centers; and Karakalpakstan, with its salt caves for therapy. Cross-border expansion into neighboring Central Asian countries with comparable natural healing resources, including thermal springs in eastern Kazakhstan (10) and the developing spa infrastructure in Kyrgyzstan, represents a long-term opportunity to establish a regional wellness tourism network.

## Discussion

This paper presents a dual argument for developing health tourism, grounded in econometric data and digital health technologies. The WLS model's finding that expanding the client base and skilled staff generate revenue growth, while overdiversification reduces efficiency, offers practical recommendations for facility managers and policymakers. The proposed voucher platform translates these findings into a practical digital architecture that integrates disparate service providers, standardizes quality assessment, and enables data-driven planning.

The link between the econometric evidence and the proposed platform is direct and structural rather than illustrative. Each operational element of the Wellness Bukhara Voucher System maps onto a specific empirical coefficient. Stage 1 (voucher purchase via mobile app) operationalizes the dominant *Users* coefficient (*β* = 18.14) by lowering search and booking costs that suppress client acquisition, particularly for international tourists who currently lack a unified discovery channel for Bukhara's wellness offerings. Stage 2 (rating- and availability-based service selection) and Stage 4 (automated analytics) jointly support the *Staff* coefficient (*β* = 15.57) by surfacing performance signals that justify and direct workforce investment toward the providers and modalities where labor productivity is highest, rather than dispersing training budgets uniformly. Stage 3 (QR-code authentication) generates the verified transactional records that the analytics layer needs in order to produce trustworthy benchmarks. Most importantly, the network-specialization logic embedded in the PPP financial model is the platform's structural answer to the negative *Services* coefficient (*β* = −31.39): rather than asking each facility to broaden its own portfolio—a strategy the data show to be revenue-destroying the platform expands the breadth of options visible to any one client through cross-referrals across specialized providers, separating *system-level diversity* from *facility-level fragmentation*. In this sense, the platform is not a generic digital wrapper around the econometric findings but an architecture engineered to amplify the two positive coefficients while neutralizing the negative one.

This approach draws parallels with, but also differs in important respects from successful implementations of digital health technologies in resource-limited settings. Mapinduzi et al. (11) demonstrated that integrated digital ecosystems in Burundi can address healthcare gaps through strategic, phased implementation; our voucher system applies similar phased principles to wellness tourism, although the user base of self-paying domestic and international tourists differs materially from the publicly funded patient population in the Burundi case, and the revenue logic of the wellness sector imposes different incentive constraints. Similarly, the synergies between telemedicine and tourism documented in rural Greece (12) and digital marketing strategies for medical tourism in Uzbekistan (5) support the feasibility of platform-based integration in regions with historical heritage, but neither prior study offers the empirical revenue model that anchors our platform design. The contribution of the present work lies precisely in coupling these two strands.

From a policy perspective, three priority areas for action are highlighted. First, investment strategies should prioritize customer acquisition infrastructure, including digital marketing, multilingual platforms, and simplified booking, over service portfolio expansion, given the strong positive coefficient for the number of users and the negative coefficient for diversification. Second, workforce development should focus on hybrid competencies that combine traditional wellness knowledge with modern wellness technologies, as the staffing ratio (15.57 million soums per employee) confirms that human capital is the sector's most productive resource. Third, digital integration should be implemented as a systemic intervention that simultaneously addresses fragmentation, quality standardization, and evidence-based planning, reflecting the integrated approach proven effective in implementing digital health services in low- and middle-income countries (11, 15). The proposed model also contributes to the broader discussion of sustainable development. The United Nations' SDG 3 increasingly recognizes preventative health services as contributing factors to population health. The creation of a digital, traceable wellness ecosystem will enable the Bukhara voucher system to generate population-level health data, integrating tourism planning and public health policy—two areas that have traditionally operated in isolation (16).

### Limitations and the boundaries of generalization

Several limitations require careful attention rather than perfunctory acknowledgment, because each constrains the inferences that can legitimately be drawn from our findings.

The most consequential constraint is *sample size and statistical power*. With 48 facility-year observations across 12 facilities, the effective sample is small, and although our leave-one-facility-out cross-validation supports coefficient stability, statistical power to detect smaller or interaction effects is limited. The coefficients reported should therefore be read as well-identified central tendencies for the Bukhara wellness sector during 2021–2024 rather than as universal parameter values; in particular, the magnitude of the diversification penalty (*β* = −31.39) is conditional on the current configuration of facilities, in which most operate at small scale with thin specialist labor markets. In larger or differently structured wellness markets, the same theoretical mechanisms may produce a smaller, or even reversed effect, and we caution against transferring the point estimate to settings whose structural conditions differ.

A second constraint is the *regional and temporal specificity of the data*. Bukhara's wellness sector combines a particular mix of UNESCO-anchored cultural tourism, indigenous balneological traditions, and post-pandemic recovery dynamics that shape both demand and supply during the 2021–2024 observation window. The early years of this window were affected by COVID-19-related disruptions to international travel, and the subsequent recovery may inflate apparent growth rates relative to long-run equilibrium trends. Generalization to other Central Asian heritage regions—Samarkand, the Fergana Valley, Karakalpakstan, eastern Kazakhstan is plausible on theoretical grounds but remains an empirical question that the present data cannot settle. Comparative panel studies across multiple heritage destinations would be required to identify which findings are sector-general and which are Bukhara-specific.

A third constraint concerns *the model's static structure*. The specification estimates average partial effects under the assumption of a stable underlying data-generating process. It does not capture intra-annual seasonality, demand shocks, or potential non-linearities—for instance, the possibility that the diversification penalty weakens or reverses above some threshold of facility scale at which specialized labor markets thicken. Dynamic panel methods, threshold regression, and high-frequency booking data would all extend the present analysis but require longer and more granular data series than were available.

A fourth constraint is that *the proposed voucher system itself remains a design proposal rather than an evaluated intervention*. The implementation considerations addressed in the previous section identify the conditions for viable deployment, but the actual effects of the platform on revenue, client acquisition, service quality, and the diversification penalty can only be established through pilot implementation and prospective evaluation. The mapping between econometric coefficients and platform stages described above is an *engineering rationale*, not an empirical demonstration that the platform will achieve those effects at the predicted magnitudes; the latter awaits a controlled rollout with pre-specified outcome measures.

Two further considerations qualify our broader claims. Digital literacy in rural wellness facilities, particularly among older practitioners working in indigenous therapeutic modalities, may be lower than the urban averages used in national digitalization indicators, creating heterogeneous adoption that could blunt the system's benefits in precisely the high-growth nature-oriented segment identified in our forecasts. And the regulatory environment in Uzbekistan continues to evolve, meaning that compliance arrangements designed at launch may need to be revisited as secondary legislation matures. Future research should therefore prioritize pilot implementation in a defined cluster of facilities with pre- and post-deployment outcome measurement, integration of real-time and seasonal analytics, comparative studies across Central Asian wellness destinations to test the generalizability of the diversification paradox, and qualitative studies of practitioner and user experience to surface adoption barriers that aggregate data cannot reveal.

Ultimately, wellness tourism in regions with historical heritage is at a critical juncture. The combination of abundant natural resources, growing global demand, and expanding digital infrastructure creates unprecedented opportunities, but only when paired with evidence-based digital mechanisms whose claims are appropriately bounded by the data that motivated them. The Bukhara model offers one way to realize this potential while remaining honest about what it does, and does not yet, demonstrate.

## Conclusion

This Perspective has argued that advancing wellness tourism in Central Asian heritage regions requires coupling econometric diagnosis with digital platform design. Using Bukhara as an empirical and design setting, the analysis shows that client acquisition and qualified staffing are the dominant levers of wellness-revenue growth, while undisciplined service expansion erodes rather than enhances facility performance. The proposed Wellness Bukhara Voucher System operationalizes these findings within a public-private partnership framework, addressing fragmentation through network specialization rather than uniform diversification.

The study's primary contribution is methodological: it demonstrates a sequence in which empirical revenue modeling constrains and informs digital architecture, rather than digital architecture being designed in isolation from economic evidence. For practice, this implies that investment in heritage wellness sectors should prioritize customer acquisition infrastructure and workforce depth over portfolio breadth, and that digital integration should be treated as a systemic rather than discrete intervention. For policy, it offers a transferable template for converting natural healing assets in heritage regions into digitally coordinated wellness economies.

Realizing this potential will require pilot implementation, comparative work across Central Asian heritage destinations, and prospective evaluation of platform effects. The Bukhara model's broader value will be determined not by the elegance of its design but by the rigor of the implementation that follows.

## Data Availability

The original contributions presented in the study are included in the article/Supplementary Material, further inquiries can be directed to the corresponding author.
